# Impact of beta‐2 microglobulin expression on the survival of glioma patients via modulating the tumor immune microenvironment

**DOI:** 10.1111/cns.13649

**Published:** 2021-05-07

**Authors:** Feng Tang, Yu‐Hang Zhao, Qing Zhang, Wei Wei, Su‐Fang Tian, Chen Li, Jie Yao, Ze‐Fen Wang, Zhi‐Qiang Li

**Affiliations:** ^1^ Brain Glioma Center & Department of Neurosurgery Zhongnan Hospital of Wuhan University Wuhan Hubei China; ^2^ Department of Pathology Zhongnan Hospital of Wuhan University Wuhan Hubei China; ^3^ Department of Biological Repositories Zhongnan Hospital of Wuhan University Wuhan Hubei China; ^4^ Department of Physiology Wuhan University School of Basic Medical Sciences Wuhan Hubei China

**Keywords:** B2M, gliomas, immunologic microenvironment, survival

## Abstract

**Aims:**

High immune cell infiltration in gliomas establishes an immunosuppressive tumor microenvironment, which in turn promotes resistance to immunotherapy. Hence, it is important to identify novel targets associated with high immune cell infiltration in gliomas. Our previous study showed that serum levels of beta‐2 microglobulin (B2M) in lower‐grade glioma patients were lower than those in glioblastoma patients. In the present study, we focused on exploring the roles of B2M in glioma immune infiltration.

**Methods:**

A large cohort of patients with gliomas from the TCGA, CGGA, and Gravendeel databases was included to explore differential expression patterns and potential roles of B2M in gliomas. A total of 103 glioma tissue samples were collected to determine the distributions of B2M protein levels by immunofluorescent assays. Kaplan‐Meier survival analysis and meta‐analysis were used for survival analysis. GO(Gene‐ontology) enrichment analysis, co‐expression analysis, KEGG(Kyoto Encyclopedia of Genes and Genomes) pathway analysis, and immune infiltration analysis were performed to explore roles and related mechanisms of B2M in glioma.

**Results:**

We found that both B2M mRNA and protein levels were abnormally upregulated in glioma samples compared with those from normal brain tissue. B2M expression was correlated with tumor grade and was downregulated in IDH1 mutant samples. Furthermore, B2M was a moderately sensitive indicator for predicting the mesenchymal molecular subtype of gliomas. Interestingly, glioma patients with lower B2M expression had remarkably longer survival times than those with higher B2M expression. Moreover, meta‐analysis showed that B2M was an independent predictive marker in glioma patients. The results of GO enrichment analysis revealed that B2M contributed to immune cell infiltration in glioma patients. In addition, results of KEGG pathway analysis and co‐expression analysis suggested that B2M may mediate glioma immune infiltration via chemokines.

**Conclusions:**

We conclude that B2M levels are critical for the survival times of glioma patients, at least in part due to mediating high immune infiltration.

## Graphical abstract

Beta‐2 microglobulin (B2M) is a potential marker for the prognosis of glioma patients. Forest plot shows that high B2M expression corresponded to a poor survival time compared to that of low B2M expression in glioma patients from three databases.

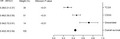

## 1 INTRODUCTION

Gliomas are the most common and lethal type of intracranial tumor. Patients with high‐grade gliomas, also known as glioblastomas (GBMs), only survive for 12–15 months despite optimal surgical and chemoradio‐therapeutic treatments.^1^, ^2^ High immune cell infiltration has been found in gliomas, during which infiltrated immune cells migrate into tumor regions to establish an immunosuppressive tumor microenvironment, which in turn promotes resistance to immunotherapy.^3^, ^4^ Hence, it is critical to identify novel targets associated with high immune cell infiltration in gliomas.

Major histocompatibility complex class I (MHC‐I) has been reported to participate in the regulation of immune escape in several tumors.^5^ Human MHC‐I molecules consist of classical human leukocyte antigen (HLA)‐A, HLA‐B, and HLA‐C, as well as non‐classical HLA‐E, HLA‐F, and HLA‐G. Each subfamily is composed of a specific MHC‐encoded polymorphic heavy chain and an invariant subunit, beta‐2 microglobulin (B2M).^6^ MHC‐I primarily plays its role through presenting antigens to T lymphocytes, which eventually leads to cytolytic damage of the presenting target cell that is mediated by CD8^+^ T cells. During the process of antigen presentation, B2M is responsible for ensuring proper loading of antigen peptides onto MHC‐I molecules and stabilizing the MHC‐I–peptide complex located on the cell surface.^7^, ^8^ In melanoma cell lines, it has been reported that B2M mutations lead to HLA‐class‐I antigen loss, which might be an early event for tumor cells progressing into the malignant phenotype.^9^ A subsequent study confirmed that B2M mutations can result in immune selection and expansion of highly aggressive melanoma clones.^10^ Moreover, high concentrations of B2M suppress the proliferation of primary tumor cells and myeloma cell lines and induce apoptosis and cell‐cycle arrest.^11^

On the contrary, several studies have indicated that B2M is upregulated in both colorectal cancer and squamous cell carcinoma to contribute to tumor progression.^12^, ^13^, ^14^ In addition, B2M protein is also a growth‐promoting molecule for human prostate, breast, and lung cancers, as well as renal cell carcinoma.^15^ It has been reported that targeting B2M with a specific anti‐B2M antibody represents a potential novel therapeutic approach for the treatment of human renal cell carcinoma, prostate cancer, and hematological malignancies.^15^, ^16^, ^17^ These findings prompted us to explore the expression patterns and potential roles of B2M in glioma patients.

In the present study, we first measured B2M mRNA and protein levels in glioma tissues and normal brain tissues from public datasets. Then, we explored the B2M expression in gliomas with respect to World Health Organization (WHO) grade, IDH1 status, and different molecular subtypes. Next, we also analyzed the correlation between B2M levels and survival times of glioma patients. Additionally, a meta‐analysis was performed to assess whether B2M could be used as a predictive marker for glioma patients. Furthermore, GO(Gene‐ontology) enrichment and immune infiltration analysis were employed to investigate the probable roles of B2M in gliomas. Finally, KEGG(Kyoto Encyclopedia of Genes and Genomes) pathway and co‐expression analysis were conducted to determine potential mechanisms of B2M in gliomas.

## 2 MATERIALS AND METHODS

### 2.1 Downloading of data from public databases

To explore the expression levels of B2M in 33 TCGA tumor types and corresponding normal tissues, we searched the online website, Gene Expression Profiling Interactive Analysis (GEPIA; http://gepia.cancer‐pku.cn/), which is an interactive web application for gene expression analysis based on 9736 tumor samples and 8587 normal samples from the TCGA and GTEx databases, respectively.^18^ The protein levels of B2M in glioma samples were obtained from the Human Protein Atlas (HPA; https://www.proteinatlas.org/). B2M expression levels and corresponding clinical data from glioma patients were downloaded from the TCGA, CGGA, and Gravendeel databases, all of which relied on the GlioVis data portal (http://gliovis.bioinfo.cnio.es/).^19^ This analysis included 1921 glioma samples (TCGA: 667 patients; CGGA: 983 patients; Gravendeel: 271 patients).

### 2.2 Glioma tissue samples

A total of 103 tumor tissue samples were collected from glioma patients with different grades and IDH1 statuses to measure B2M protein levels. All samples were obtained from Zhongnan Hospital of Wuhan University and were diagnosed according to the 2016 WHO classification guidelines. This study was approved by the Ethics Committee of Zhongnan Hospital of Wuhan University (no. 2019048).

### 2.3 Meta‐analysis

A literature search was performed to identify published studies related to B2M and prognosis of gliomas across the PubMed, Embase, and Web of Science databases. Considering that there was only one study based on the TCGA and CGGA databases referring to B2M levels and survival times of lower‐grade gliomas (LGGs) patients, we performed a meta‐analysis to assess the predictive significance of B2M in gliomas by using data from the prior three datasets. The meta‐analysis was completed using STATA 15.1 software.

### 2.4 Differential expression analysis, GO enrichment analysis, and KEGG analysis

Differential expression analysis, GO enrichment analysis, and KEGG analysis were performed through the GlioVis data portal. First, the microarray data from the Gravendeel database were divided into low and high expression groups according to B2M expression levels. Differentially expressed genes between the low and high B2M expression groups were selected when |logFC| ≥ 2 was combined with a *p* < 0.05. Subsequently, we analyzed the related biological processes and KEGG pathways to gain insight into the roles and related pathways of B2M in gliomas according to the differentially expressed genes between the low and high B2M expression groups.

### 2.5 Immune infiltration analysis

Immune infiltration analysis was performed through the TIMER web server, which is a comprehensive resource for systematical analysis of immune infiltrates across diverse cancer types (https://cistrome.shinyapps.io/timer/). The abundances of six immune infiltrates (B cells, CD4^+^ T cells, CD8^+^ T cells, neutrophils, macrophages, and dendritic cells) were estimated by the TIMER algorithm.^20^ We evaluated the correlation of B2M expression with the abundance of immune cells and the prognostic value of B2M in gliomas patients with different abundances of immune cells.

### 2.6 Immunofluorescence assay

Briefly, the paraffin sections of glioma tissues were dewaxed and dewatered with gradient alcohol, after which antigen repair was performed. Then, the samples were washed three times with PBST. Next, glioma tissues were sealed with 10% BSA(Bovine serum albumin) in a 37°C wet box for 30 min. Thereafter, the samples were incubated with a B2M antibody (1:100, 13511‐1‐AP, Proteintech) in a 37°C wet box for 30 min, after which the samples were washed three times with PBST. Then, these samples were incubated with IgG/FITC antibody (1:200, bs‐0293G‐FITC; Bioss) for 1 h at 37°C. Finally, these samples were stained with DAPI for 5 min at room temperature, and images were acquired using a fluorescent‐microscope imaging system. The mean fluorescence value (mean fluorescence value = integrated density/area) of each immunofluorescent image was measured by the ImageJ software. The results we got were just a “semi‐quantitative” B2M protein levels. After that, all glioma samples were divided into weak, moderate, or strong fluorescent intensity according to the mean fluorescence value of each sample. Statistical results of immunofluorescent intensities of B2M proteins in glioma samples were presented in Tables 1 and 2.

**TABLE 1:** Statistical results of immunofluorescence intensity of B2M protein in LGGs and GBMs

Intensity	LGGs	GBMs	Total
Weak	29 (28.2%)	6 (5.8%)	35 (34%)
Moderate	26 (25.2%)	14 (13.6%)	40 (38.8%)
Strong	10 (9.7%)	18 (17.5%)	28 (27.2%)
Total	65 (63.1%)	38 (36.9%)	103 (100%)

Note

LGGs (*n* = 65), lower‐grade gliomas; GBMs (*n* = 38), glioblastomas.

Abbreviations: B2M, beta‐2 microglobulin; GBM, glioblastoma; LGG, lower‐grade glioma.

**TABLE 2:** Statistical results of immunofluorescence intensity of beta‐2 microglobulin protein in IDH1 wild‐type and IDH1 mutant gliomas

Intensity	IDH1 wild type	IDH1 mutant	Total
Weak	8 (7.8%)	27 (26.2%)	35 (34%)
Moderate	23 (22.3%)	17 (16.5%)	40 (38.8%)
Strong	26 (25.2%)	2 (2%)	28 (27.2%)
Total	57 (55.3%)	46 (44.7%)	103 (100%)

Note

IDH1 wild‐type gliomas (*n* = 57); IDH1 mutant gliomas (*n* = 46).

### 2.7 Statistical analysis

SPSS 23.0, GraphPad Prism 8.0, and ImageJ software were used for statistical analysis. A *p* < 0.05 was considered statistically significant. In this study, Shapiro–Wilk test was the used to assess the variables distribution. Student's *t* test or one‐way ANOVA test was performed to analyze data that follows a normal distribution. Otherwise, Mann–Whitney test was used to evaluate the data that do not exhibit a normal/Gaussian distribution.

## 3 RESULTS

### 3.1 B2M is aberrantly expressed in glioma samples

**FIGURE 1:**
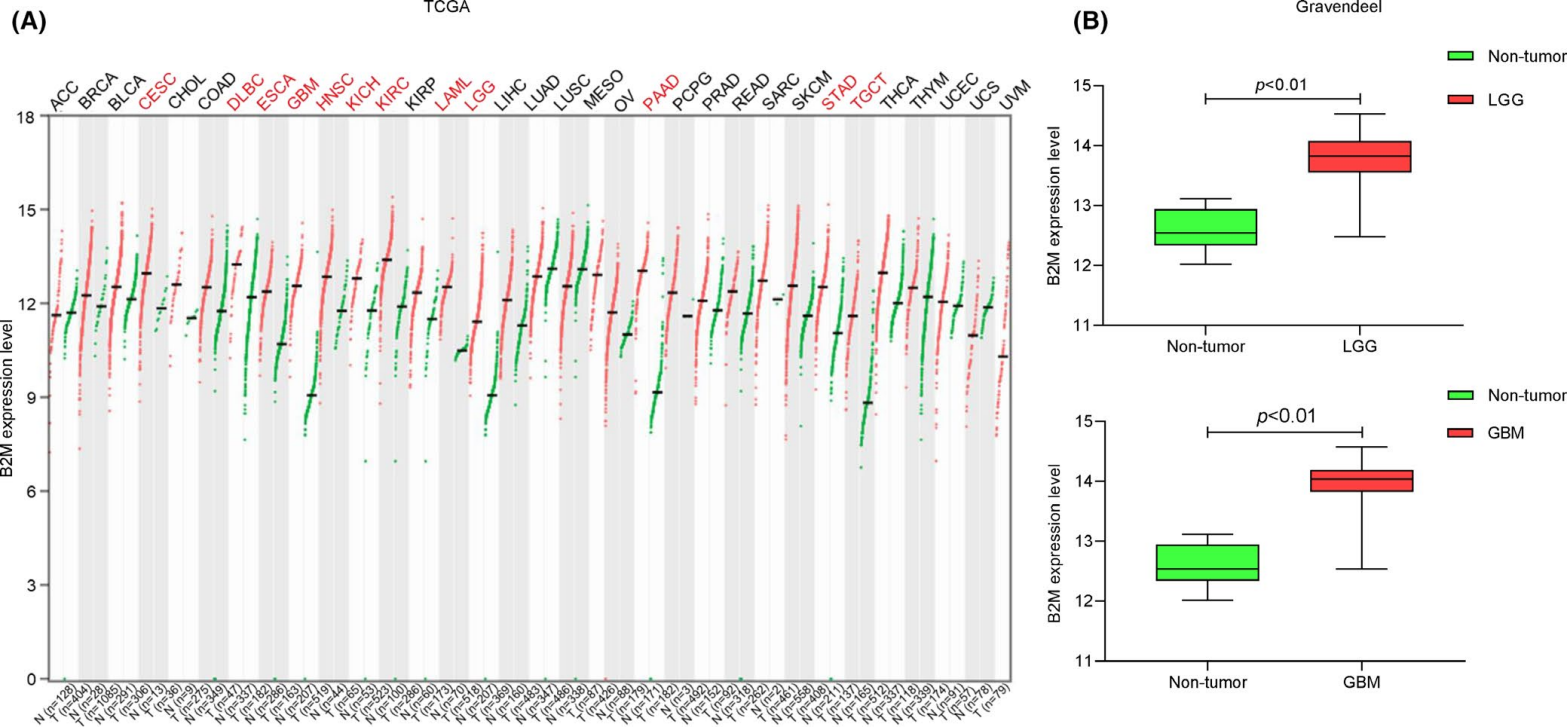
Beta‐2 microglobulin (B2M) mRNA levels in tumors and corresponding normal tissues. (A) B2M mRNA levels in 33 tumor types in the The Cancer Genome Atlas dataset. (B) B2M expression in lower‐grade gliomas (LGGs) and glioblastomas (GBMs) in the Gravendeel dataset

**FIGURE 2:**
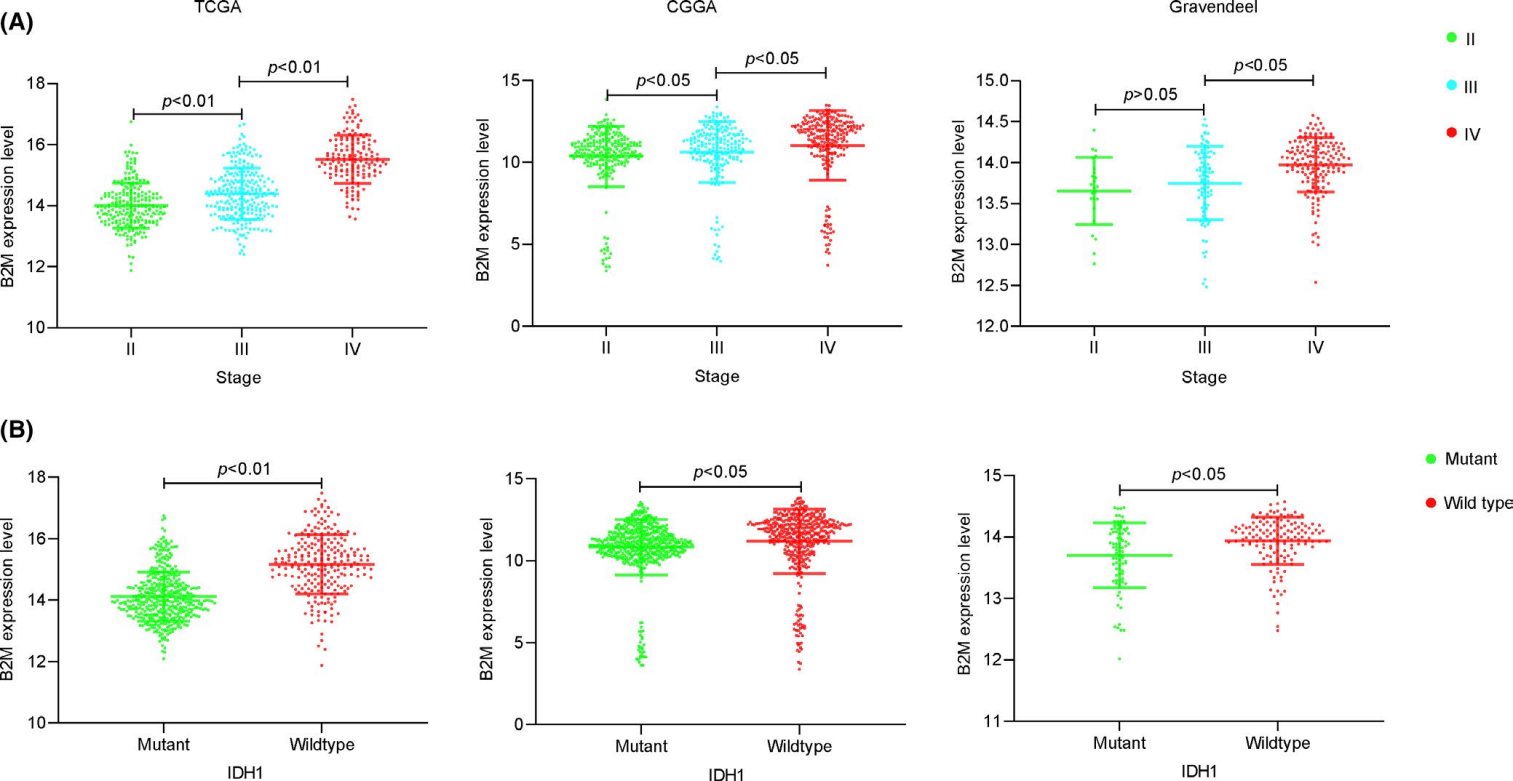
Beta‐2 microglobulin (B2M) mRNA levels in different grades and IDH1 statuses of gliomas. (A) B2M mRNA levels in different grades of gliomas B. B2M mRNA levels in different IDH1 statuses of gliomas

Firstly, the RNA‐sequencing data of all 33 TCGA tumor types and corresponding normal samples were obtained from TCGA and GTEx databases, respectively, to explore expression patterns of B2M in tumors. As shown in Figure 1A, upregulated B2M mRNA expression was observed in LGGs, GBMs, cervical squamous cell carcinoma and endocervical adenocarcinoma, lymphoid neoplasm diffuse large B‐cell lymphoma, esophageal carcinoma, head and neck squamous cell carcinoma, kidney chromophobe, kidney renal clear cell carcinoma, acute myeloid leukemia, pancreatic adenocarcinoma, stomach adenocarcinoma, and testicular germ cell tumors compared with that in corresponding normal tissues. The results of microarray data from the Gravendeel database also showed that B2M mRNA expression was significantly increased in both LGGs and GBMs (Figure 1B). Next, we explored the correlation between B2M mRNA expression and tumor grade or IDH1 phenotype. As shown in Figure 2A, B2M levels were positively correlated with tumor grade, and expression of B2M in grade IV (also known as GBMs) was higher than that in grade II or grade III (also known as LGGs). Furthermore, decreased B2M expression was found in the IDH1 mutant type compared with that in the IDH1 wild type (Figure 2B).

**FIGURE 3:**
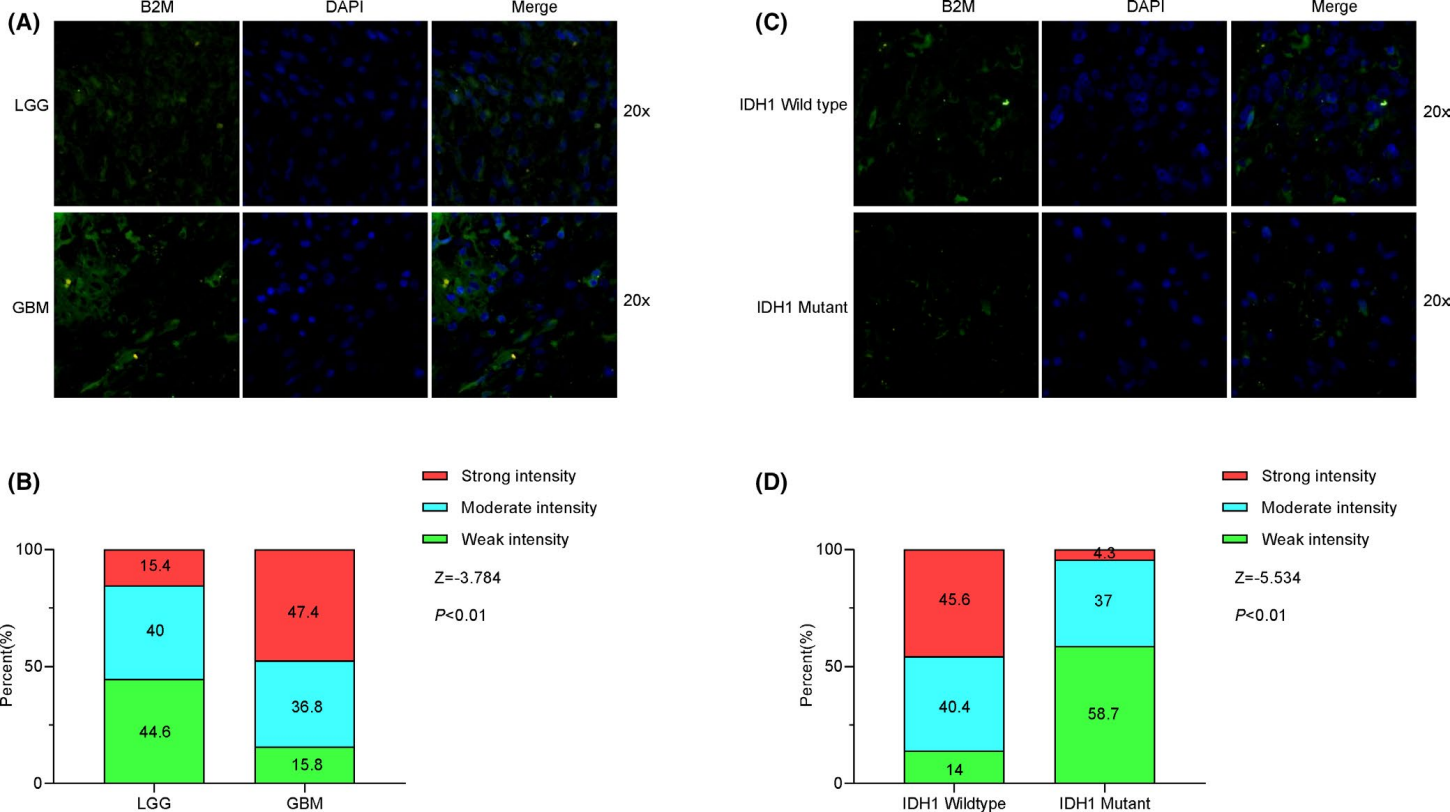
Beta‐2 microglobulin (B2M) protein levels in tumor tissues from glioma patients. (A, B) B2M protein levels in lower‐grade glioma (LGG) and glioblastoma (GBM) tissues. (C, D) B2M protein levels in different IDH1 status tissues

Moreover, results from the HPA database also showed that B2M protein expression was higher in glioma tissues, especially in GBMs, compared with that in normal tissues (Figure S1A). And the B2M protein was mainly distributed in the membrane and cytoplasm in glioma cells (Figure S1B). To validate these findings, we detected B2M protein levels in 103 glioma tissues by immunofluorescence assay. The fluorescent intensity of B2M protein in each sample was classified as weak, moderate, or strong, such that a weak intensity was indicative of low B2M expression. As shown in Figure 3A,B, the results of a Mann‐Whitney U test showed that the expression levels of B2M in LGGs (weak intensity 44.6%; moderate intensity 40%; strong intensity 15.4%) and GBMs (weak intensity 15.8%; moderate intensity 36.8%; strong intensity 47.4%) were significantly different from one another (*Z* = −3.784, *p* < 0.01). Compared with those in LGGs, there were stronger fluorescent signals in GBMs. In addition, B2M levels in IDH1 mutant glioma (weak intensity 58.7%; moderate intensity 37%; strong intensity 4.3%) samples were lower than those in IDH1 wild‐type (weak intensity 14%; moderate intensity 40.4%; strong intensity 45.6%) samples(Figure 3C,D, *Z* = −5.534, *p* < 0.01).

### 3.2 B2M is a moderately sensitive marker for mesenchymal molecular subtype gliomas

**FIGURE 4:**
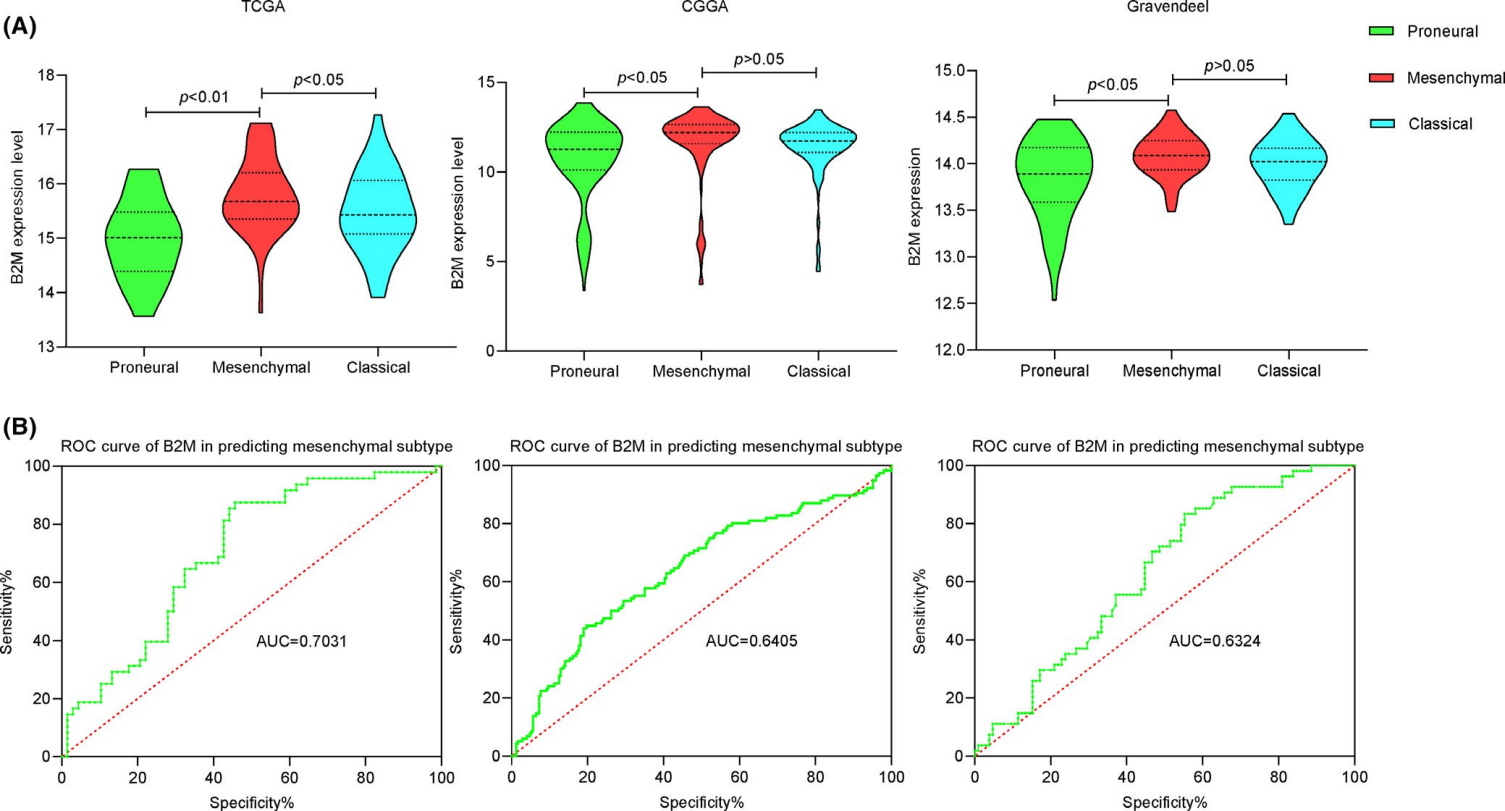
The expression pattern and predictive role of beta‐2 microglobulin (B2M) in the mesenchymal molecular subtype of gliomas. (A) B2M mRNA levels in different molecular subtypes of gliomas across three databases. (B) ROC curves of B2M in predicting the mesenchymal subtype of gliomas in three databases

To further explore the expression patterns of B2M in gliomas, we evaluated the distribution of B2M in different molecular subtypes. Compared with that in the proneural molecular subtype, B2M expression was higher in the mesenchymal molecular subtype among the three glioma databases. In addition, there was also a tendency for B2M expression in the mesenchymal molecular subtype to be increased compared with that in the classical molecular subtype (Figure 4A). To validate this result, we performed receiver operating characteristic curve (ROC) analysis for B2M expression in the mesenchymal molecular subtype of gliomas. Results showed that the areas under the curves(AUCs) varied from 63.24% to 70.31% among the three databases, indicating that B2M possessed a moderate sensitivity and specificity for predicting the mesenchymal molecular subtype of gliomas (Figure 4B).

### 3.3 B2M is a potential prognosis marker in glioma patients

**FIGURE 5:**
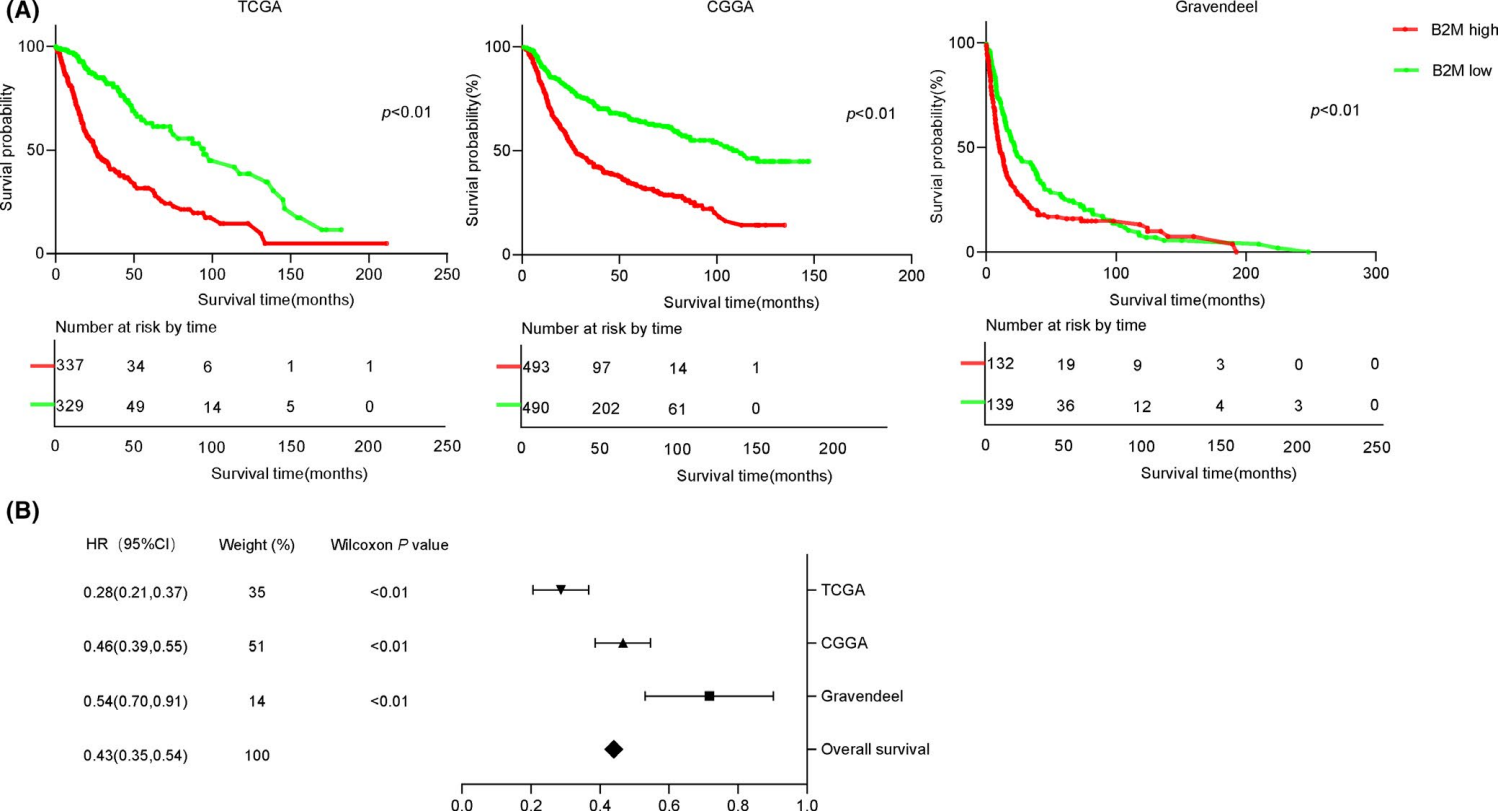
Beta‐2 microglobulin (B2M) is a potential marker for the prognosis of glioma patients. A. Survival probabilities of glioma patients in the high and low B2M groups across three databases. B. Forest plot shows that high B2M expression corresponded to a poor survival time compared to that of low B2M expression in glioma patients from three databases

Next, we further investigated the clinical significance of B2M in glioma patients. As shown in Figure 5A, results of Kaplan‐Meier analysis showed that patients with lower B2M expression had remarkably better overall survival than those with higher B2M expression. Because there was only one study based on TCGA and CGGA databases referring to B2M levels and survival times of LGG patients, we also performed a meta‐analysis by using data from the former three datasets.^21^ The pooled hazard ratio along with the 95% confidence interval for the association between low B2M expression and overall survival in 1921 cases of glioma patients was 0.43 (0.35–0.54), with no significant heterogeneity among the three datasets(Figure 5B). Taken together, these results revealed that B2M might function as an independent prognostic indicator for glioma patients.

### 3.4 High expression of B2M is related to immune cell infiltration in glioma samples

**FIGURE 6:**
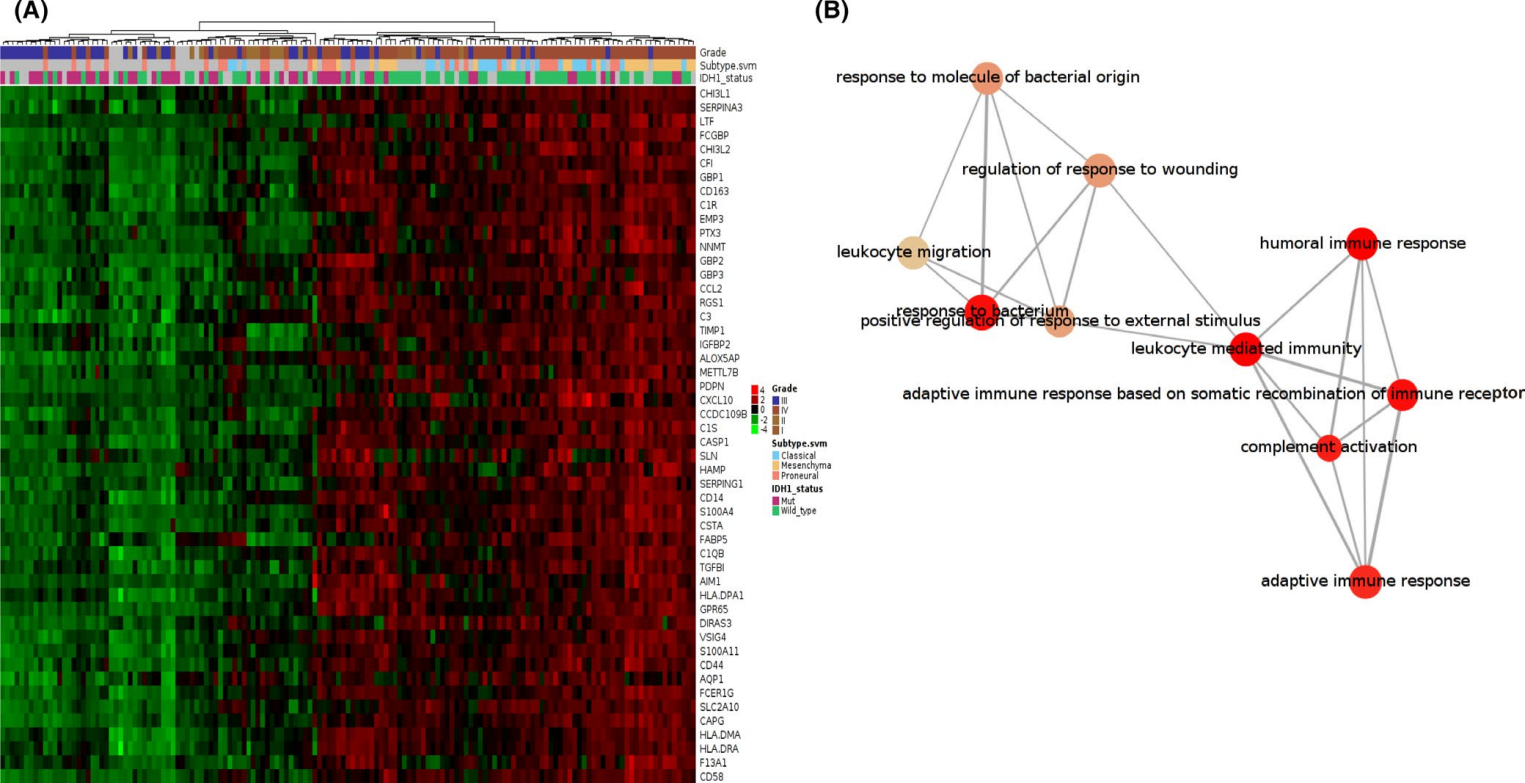
Beta‐2 microglobulin (B2M) is related to migration of immune cells in gliomas. (A) The top‐50 genes related to B2M expression. B. The top‐10 GO terms in the B2M‐associated biological processes

**FIGURE 7:**
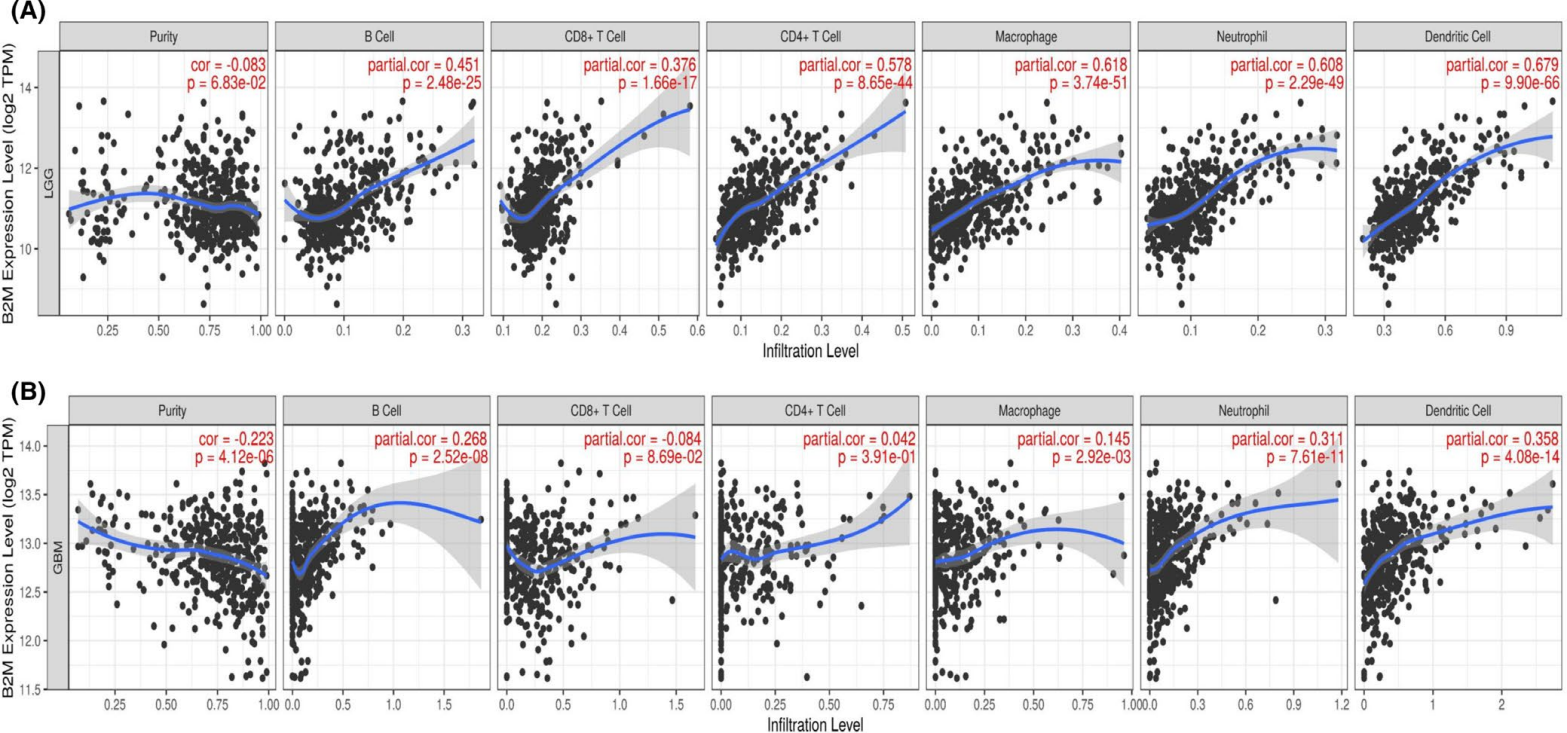
Beta‐2 microglobulin (B2M) is associated with immune cell infiltration. (A, B) Relationship between B2M expression and levels of immune infiltration in lower‐grade gliomas (LGGs) and glioblastomas (GBMs)

The microarray data from the Gravendeel dataset were used to perform GO enrichment analysis via the Gliovis tool. There were 183, and 18 genes were positively and negatively related, respectively, to B2M expression. And the Figure 6A shows the top‐50 differentially expressed genes. The top‐10 GO terms in biological processes suggested that B2M was mainly involved in regulating immune response in gliomas. Interestingly, our results indicated that B2M may also modulate leukocyte migration and leukocyte‐mediated immunity (Figure 6B). To determine which leukocyte cell type was associated with B2M‐mediated immune responses, we divided samples into high and low B2M expression groups and then analyzed immune infiltration in gliomas based on the TIMER dataset. Tumor purity has been reported as an underlying key factor in gliomas, and low‐purity gliomas are enriched with immune cells.^22^ After adjusting for the influence of glioma purity, we found that high expression of B2M in LGGs was correlated with high levels of infiltration of B cells, CD8^+^ T cells, CD4^+^ T cells, macrophages, NK cells, and dendritic cells, while low expression of B2M yielded opposite results (Figure 7A). However, there was no obvious correlation between B2M expression and CD8^+^ T or CD4^+^ T cells infiltration in GBMs (Figure 7B).

In addition, our further results revealed that the copy number of the B2M gene also affected infiltration levels mediated by immune cells. As shown in Figure S2A,B, deep deletion or arm‐level gain of the B2M gene did not influence infiltration levels in either LGGs or GBMs. However, arm‐level deletion in the B2M gene decreased infiltration levels of CD4^+^ T cells, macrophages, and neutrophils in LGGs. Interestingly, arm‐level deletion in the B2M gene also decreased infiltration levels of CD8^+^ T cells in GBMs.

### 3.5 B2M mediates glioma immune infiltration via chemokines

**FIGURE 8:**
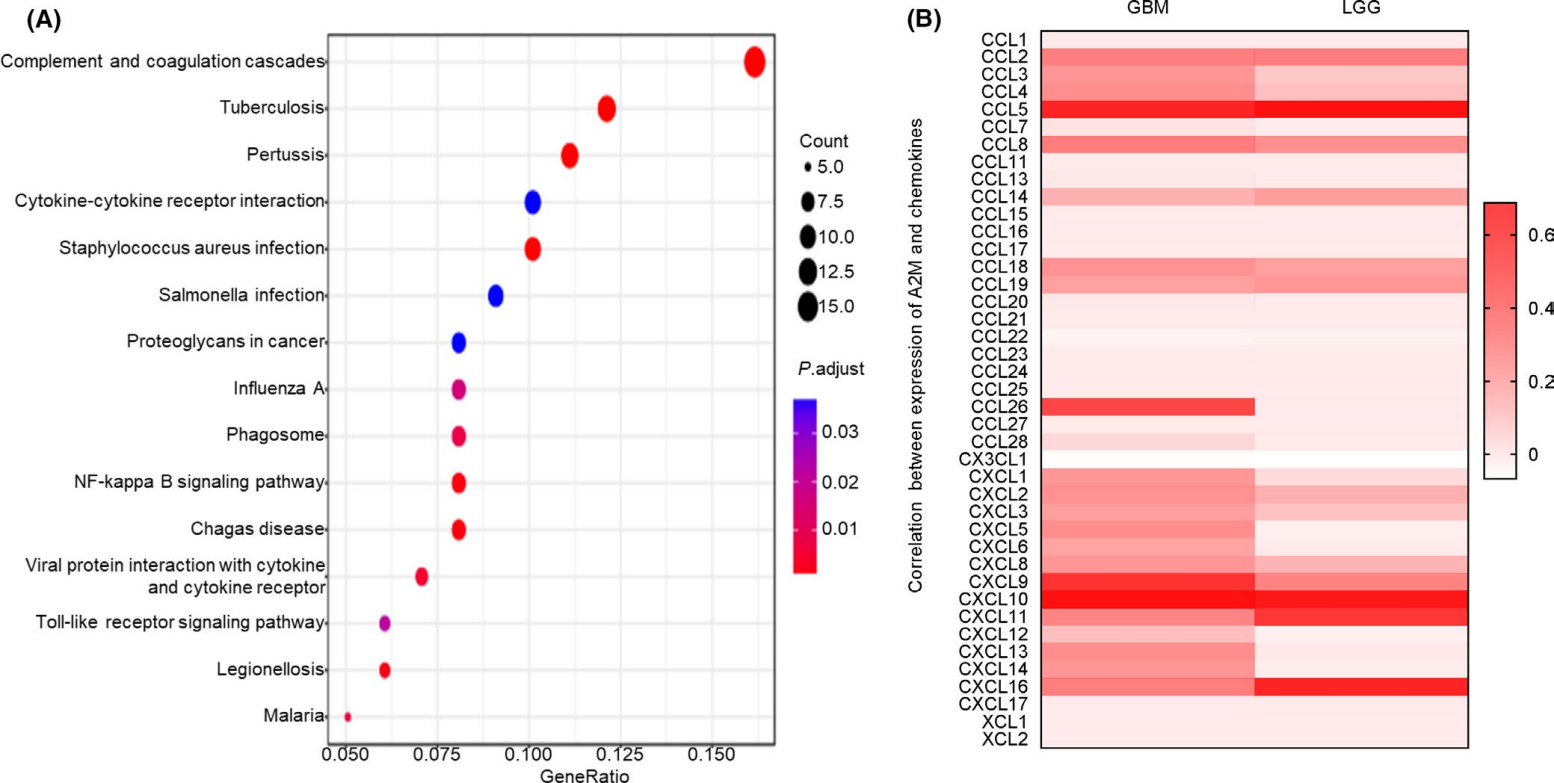
Beta‐2 microglobulin (B2M) regulates immune infiltration through chemokines in gliomas. (A) B2M‐associated pathways in gliomas. (B) Co‐expression of chemokines and B2M in gliomas

To further gain insight into the mechanisms of B2M in gliomas, we performed KEGG pathway analysis. As shown in Figure 8A, B2M was involved in cytokine‐cytokine receptor interactions, proteoglycans in cancer, the NF‐kappa‐B signaling pathway, and the Toll‐like receptor signaling pathway. Thus, we speculated that B2M might mediate glioma immune infiltration via chemokines. To test this hypothesis, co‐expression of B2M and related chemokines was analyzed based on TISIDB (http://cis.hku.hk/TISIDB/), which is a web portal for tumor and immune‐system interactions.^23^ There were several chemokines that were highly correlated with B2M expression (Figure 8B). Interestingly, the two chemokines most related to B2M expression were CXCL10 and CCL5 in both LGGs and GBMs (Figures S2C,D). Therefore, we explored the impact of immune infiltration in gliomas. The results suggested that LGG patients with lower immune cell infiltration had longer survival times compared to those with higher immune cell infiltration (Figure S3A; Table 3). Nevertheless, there was no significant correlation between immune infiltration and survival time in GBM patients, except for in dendritic cells (Figure S3B).

**TABLE 3:** Univariate analysis of the correlation among B2M expression, immune infiltration, and OS in patients with gliomas

Glioma	Variable	*p*
LGGs	B cell	4.25E−05
LGGs	CD8^+^ T cell	0.009535293
LGGs	CD4^+^ T cell	0.000460305
LGGs	Macrophage	9.20E−06
LGGs	Neutrophil	5.83E−06
LGGs	Dendritic cell	0.000634174
LGGs	B2M	0.000118692
GBMs	B cell	0.105243251
GBMs	CD8^+^ T cell	0.805152757
GBMs	CD4^+^ T cell	0.629122347
GBMs	Macrophage	0.755368558
GBMs	Neutrophil	0.741002234
GBMs	Dendritic cell	0.001616954
GBMs	B2M	0.003233862

Note

LGGs (*n* = 505), lower‐grade gliomas; GBMs (*n* = 523), glioblastomas.

Abbreviations: B2M, beta‐2 microglobulin; GBM, glioblastoma; LGG, lower‐grade glioma.

## 4 DISCUSSION

In this study, we detected B2M mRNA levels in 33 tumor types and corresponding normal samples. Increased B2M expression was observed in 12 out of 33 tumor types compared with that in corresponding normal tissues. In colorectal cancer and squamous cell carcinoma, B2M expression was upregulated, which is consistent with findings from previous studies.^12^, ^14^ In glioma samples, we found that B2M levels in both LGG and GBM samples were higher than those in normal brain tissues. Results from the Gravendeel database confirmed this finding. Subsequent HPA dataset results also indicated that B2M protein was upregulated in glioma patients. Furthermore, B2M protein was mainly distributed in the plasma membrane and cytoplasm in glioma cells. To exclude the influence of patient ethnicity, data from the CGGA database were also selected for further analysis. The results revealed that B2M expression was positively correlated with glioma grade and remarkably varied in different IDH1 status groups. Immunofluorescent assays of glioma samples we collected also showed a similar result. Additionally, we also evaluated the distribution of B2M in different molecular subtypes of gliomas. There was a tendency for B2M expression in the mesenchymal molecular subtype to be higher compared with that in the proneural or classical molecular subtype. The results of ROC analysis indicated that B2M possessed a moderate sensitivity and specificity for predicting the mesenchymal molecular subtype of gliomas. Previous studies have shown that B2M serum levels are also abnormal in several tumors.^24^, ^25^ Our previous study also showed that serum levels of B2M in LGG patients were lower than those in GBM patients, while levels of B2M in the IDH1 wild type were higher than those in the IDH1 mutant.^26^ Therefore, expression patterns of B2M in serum are similar to those in glioma tissues. Considering the existence of the blood‐brain barrier in the central nervous system, we conclude that the combined detection of B2M levels in tumor tissues, cerebrospinal fluid, and serum might enable optimal specificity for the diagnoses of different molecular subtypes and clinical stages of gliomas. Apart from the B2M was abnormally upregulated, other recent reported molecules such as NUSAP1, Paxillin, CAVIN1, and PARP9 also overexpressed in glioma tissues.^27^, ^28^, ^29^, ^30^ Thus, we speculate that it will improve the diagnostic accuracy of gliomas in combination with these molecules.

In our present study, Kaplan–Meier analysis showed that patients with lower B2M expression exhibited remarkably better overall survival than those with higher B2M expression. We also performed a meta‐analysis using the data from the former three datasets.^21^ Results showed that B2M to be an independent predictive marker in glioma patients. Taken together, these results revealed that B2M might function as an independent prognostic indicator for gliomas. In squamous cell carcinoma and breast cancer, tissue B2M levels have also been shown to be independent prognostic factors predictive of overall survival.^13^, ^31^ However, there is no significant correlation between B2M expression and survival in non‐small cell lung cancer.^32^ Collectively, these studies indicate that the potential of B2M as an independent prognostic indicator for cancers is tissue‐dependent.

The results of GO enrichment analysis in terms of biological processes in our present study suggested that B2M was involved in modulating leukocyte migration and leukocyte‐mediated immunity. Further immune infiltration analyses showed that high B2M expression presented high immune infiltration of B cells, CD8^+^ T cells, CD4^+^ T cells, macrophages, neutrophil, and dendritic cells in LGGs. However, expression of B2M was positively related to infiltration of B cells, macrophages, neutrophil, and dendritic cells in GBMs. In addition, our further research found that the copy number of the B2M gene could also affect the infiltration levels mediated by immune cells. Interestingly, arm‐level deletion of the B2M gene decreased infiltration levels of CD8^+^ T cells in GBMs, which was inconsistent with the results shown in Figure 7B that there was no evident correlation between B2M expression and infiltration levels of CD8^+^ T cells. Therefore, the relationship between B2M expression and infiltration levels of CD8^+^ T cells requires further investigation. Our present study is distinct from previous studies that have mainly focused on exploring the role of B2M in tumor growth, invasion, and apoptosis, or on the role of B2M as a part of MHC‐I for antigen presentation.^9^, ^13^, ^15^, ^17^, ^33^, ^34^, ^35^ For example, knockdown of B2M expression has been shown to inhibit tumor cell migration and invasion in oral cavity squamous cell carcinoma. In addition, B2M overexpression has been shown to support bone metastasis in human prostate, breast, lung, and renal cancer cells via regulating the epithelial‐to‐mesenchymal transition of tumor cells. In melanoma, it has been reported that B2M mutations lead to HLA‐class‐I antigen loss, which in turn promotes tumor cells to progress into the malignant phenotype. In contrast, our present study focused on exploring the roles of B2M in glioma immune infiltration.

After determining the roles of B2M in glioma immune infiltration in our present study, we next performed KEGG pathway analysis to explore the related mechanisms. We found that the cytokine signaling pathway, NF‐kappa B signaling pathway, and Toll‐like receptor signaling pathway were involved in B2M‐related pathways in glioma samples. Considering that leukocyte chemotaxis is mediated by chemokines, we next evaluated the correlation between B2M expression and chemokine expression. We found that there were several chemokines highly correlated with B2M expression. Among these chemokines, the two chemokines most related to B2M expression were CXCL10 and CCL5 in both LGGs and GBMs. In gliomas, CXCL10 upregulation may promote the recruitment of T cells.^36^ Higher expression of CCL5 protein has also been detected in glioma tissues and is associated with glioma‐associated microglial activation.^37^ In addition, other chemokines that are highly correlated with B2M expression also participate in different roles in glioma immune infiltration. Finally, in our present study, we explored the influence of immune infiltration on the survival times of glioma patients. We found that glioma patients with lower immune cell infiltration showed longer survival times compared to those with higher immune cell infiltration. Thus, we conclude that B2M might decrease the survival times of glioma patients, at least in part due to mediating high immune infiltration.

Recent years, patients with gliomas or other tumors have indeed benefited from the immunotherapy. Immune checkpoint blockade has been regarded as emerging approach to cancer treatment.^38^ However, high immune cell infiltration mediated immunosuppressive tumor microenvironment generally results in resistance to immunotherapy.^4^ Therefore, many researches have focused on identifying novel targets associated with high immune cell infiltration in gliomas. For example, high COPB2 expression was closely linked to higher immune cell infiltration in gliomas.^39^ Tumor‐associated antigen URGCP was proved as a potential immunotherapeutic for GBM patients.^40^ High levels of costimulatory checkpoint SLAMF8 was involved in aggravated immunosuppression.^41^ Our present study also showed that high B2M expression could mediate high immune infiltration in gliomas. In human prostate cancer cells, it has been reported that blockade of downstream signaling of B2M induces tumor cell apoptosis.^42^ Takeo et al. reported that specific antibodies against B2M have remarkable tumoricidal activities in human renal cell carcinoma by targeting B2M‐mediated signaling.^15^ In an immunocompetent spontaneous prostate cancer mouse model, B2M antibody was able to prevent tumor growth.^16^ Additionally, B2M monoclonal antibodies have been shown to exhibit therapeutic efficacy and low toxicity in human‐like myeloma mouse models, which express mature and functional human B2M in murine organs and present high levels of circulating human B2M derived from human myeloma cells.^43^ These findings suggest that B2M antibodies may represent promising treatments for cancer therapy. Therefore, our subsequent study will focus on exploring the effects of B2M inhibition on glioma immune cell infiltration and survival times in glioma patients.

## 5 CONCLUSIONS

In this study, we found that both B2M mRNA and protein levels were increased in glioma tissue samples compared to those in normal tissue samples. Glioma patients with high levels of B2M expression had a poor overall survival compared to that of glioma patients with comparatively lower levels of B2M expression. The results of GO enrichment and KEGG pathway analysis revealed that B2M regulated immune infiltration via chemokines in gliomas. Moreover, glioma patients with lower immune cell infiltration showed longer survival times compared to those with higher immune cell infiltration. Thus, we conclude that B2M might decrease the survival times of glioma patients, at least in part due to mediating high immune infiltration.

## ABBREVIATIONS

All abbreviations and full names are presented in Table 4.

**TABLE 4:** Abbreviations and full names

Abbreviations	Full names
B2M	Beta‐2 microglobulin
TCGA	The Cancer Genome Atlas
CGGA	Chinese Glioma Genome Atlas
GTEx	Genotype‐Tissue Expression Project
MHC‐I	Major histocompatibility complex class I
HLA	Human leukocyte antigen
GBMs	Glioblastomas
LGGs	Brain lower‐grade gliomas
ACC	Adrenocortical carcinoma
BLCA	Bladder urothelial carcinoma
BRCA	Breast invasive carcinoma
CESC	Cervical squamous cell carcinoma and endocervical adenocarcinoma
CHOL	Cholangio carcinoma
COAD	Colon adenocarcinoma
DLBC	Lymphoid neoplasm diffuse large B‐cell lymphoma
ESCA	Esophageal carcinoma
HNSC	Head and neck squamous cell carcinoma
KICH	Kidney chromophobe
KIRC	Kidney renal clear cell carcinoma
KIRP	Kidney renal papillary cell carcinoma
LAML	Acute myeloid leukemia
LIHC	Liver hepatocellular carcinoma
LUAD	Lung adenocarcinoma
LUSC	Lung squamous cell carcinoma
MESO	Mesothelioma
OV	Ovarian serous cystadenocarcinoma
PAAD	Pancreatic adenocarcinoma
PCPG	Pheochromocytoma and paraganglioma
PRAD	Prostate adenocarcinoma
READ	Rectum adenocarcinoma
SARC	Sarcoma
SKCM	Skin cutaneous melanoma
STAD	Stomach adenocarcinoma
TGCT	Testicular germ cell tumors
THCA	Thyroid carcinoma
THYM	Thymoma
UCEC	Uterine corpus endometrial carcinoma
UCS	Uterine carcinosarcoma
UVM	Uveal melanoma
AUCs	Areas under the curves
ROC	Receiver operating characteristic curve
CI	Confidence interval
OS	Overall survival
GEPIA	Gene Expression Profiling Interactive Analysis
HPA	Human Protein Atlas
WHO	World Health Organization
GO	Gene‐ontology
KEGG	Kyoto Encyclopedia of Genes and Genomes
BSA	Bovine serum albumin

## CONFLICT OF INTEREST

The authors declare no conflict of interest.

## Supporting information

Supplementary Material

## Data Availability

The data that support the findings of this study are available from the corresponding author upon reasonable request.
