# Supplementary material for: Impact of beta‐2 microglobulin expression on the survival of glioma patients via modulating the tumor immune microenvironment
Source: CNS Neurosci Ther. 2021 May 7;27(8):951–62. doi: 10.1111/cns.13649 (PMC8265948; doi:10.1111/cns.13649)
Supplement: Supplementary file 1 — Supplementary Material [file CNS-27-951-s001.docx]

**SUPPLEMENTARY FIGURE LEGENDS**

**
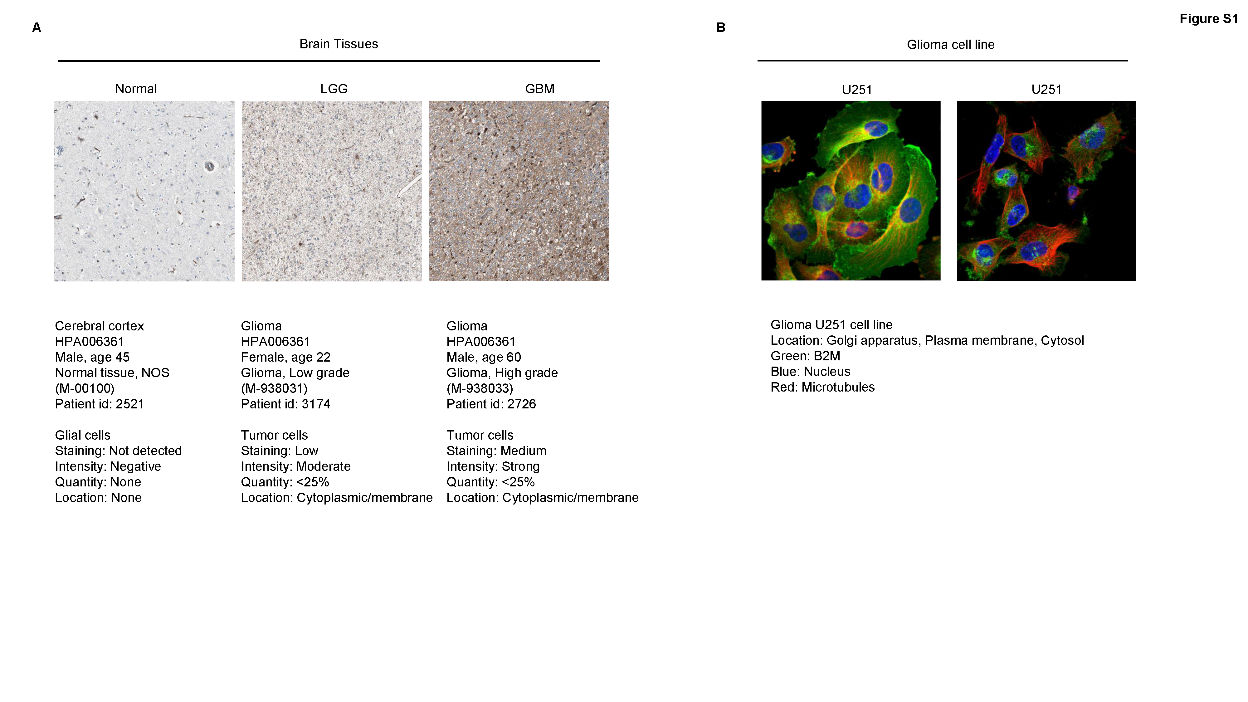
**

**Figure S1** Expression patterns of B2M protein in glioma tissues and cell lines. A. B2M protein expression in normal and glioma tissues based on the HPA database. B. Distribution of B2M protein in the glioma U251 cell line.


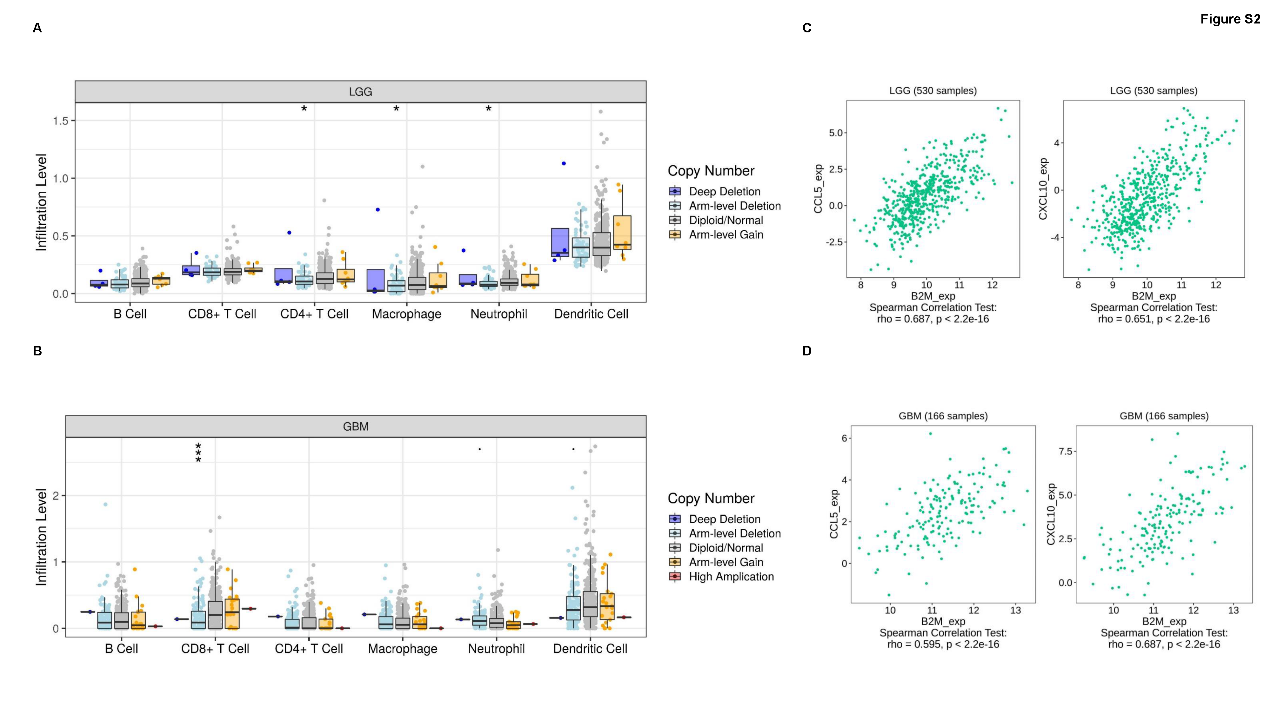


**Figure S2** Effects of the copy number of B2M on glioma immune infiltration. A–B. Relationship between B2M copy number and levels of immune infiltration in LGGs and GBMs. C–D. Both CCL5 and CXCL10 expression levels are positively correlated with B2M expression.


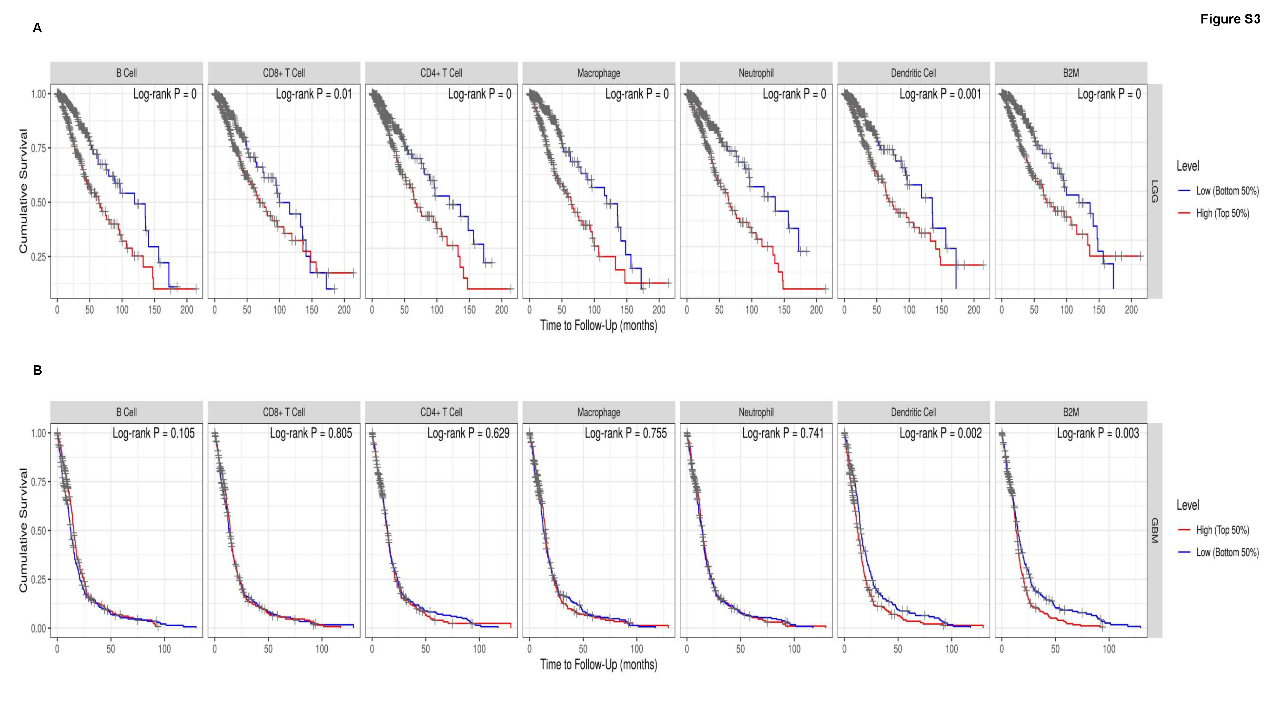


**Figure S3** A–B. The impact of immune infiltration on survival times of glioma patients.

**ORIGINAL IMMUNOFLUORESCENT IMAGES**
